# Short-Term Effects of Platelet-Rich Plasma on the Clinical Outcomes of Dental Autotransplantation in Teeth with Complete and Incomplete Root Development: A Randomized Cohort Study

**DOI:** 10.7150/ijms.113282

**Published:** 2025-07-28

**Authors:** Aysenur Genc, Betul Gedik, Mehmet Ali Erdem, Abdulkadir Burak Cankaya

**Affiliations:** 1 Boston University Henry M Goldman School of Dental Medicine, Boston, USA.; 2 Department of Oral and Maxillofacial Surgery, Istanbul University, Istanbul, Turkiye.

**Keywords:** dental autotransplantation, platelet-rich plasma, root development, clinical outcomes, oral surgery

## Abstract

**Background:** Dental autotransplantation, the surgical relocation of a tooth within the same individual, offers a valuable alternative to implants for preserving alveolar bone integrity and achieving functional restoration. This study aimed to assess the short-term impact of platelet-rich plasma (PRP) on clinical outcomes in autotransplanted teeth with fully and partially developed roots.

**Methods:** A total of 20 patients, aged 18-25, were randomly assigned to either a PRP or non-PRP group, with subgroups based on root development stage. Key outcomes—including tooth vitality, periodontal probing depth, mobility, pain, and root resorption—were evaluated at 1 week, 1, 3, 6, and 12 months post-transplant.

**Results:** Findings indicated no statistically significant differences between PRP-treated and control groups across all outcomes, suggesting limited PRP efficacy in enhancing short-term outcomes in mature teeth with developed roots.

**Conclusions:** These results underscore the importance of root maturity and atraumatic surgical technique in autotransplantation success, while highlighting that PRP may not significantly affect outcomes in teeth with completed root development.

## Introduction

Dental autotransplantation is a surgical procedure that involves transplantation a tooth from one site to another within the same individual. This technique is generally indicated when a tooth in one area is untreatable and a suitable donor tooth is available. Historically, autotransplantation was attempted until the 1950s but faced numerous failures due to limited understanding of biological foundations and inadequate clinical techniques 1, 2. However, with advancements in dental science and surgical protocols, this technique has gained renewed interest and now demonstrates promising success in carefully selected cases.

Autotransplantation is especially valuable for young individuals experiencing early tooth loss, where appropriate case selection plays a critical role in achieving positive outcomes. The primary indications for this procedure include untreatable teeth, teeth lost due to periodontal disease, root fractures, congenital tooth absence, trauma-related tooth loss, and the presence of impacted or ectopic teeth 3, 4. Despite its advantages, autotransplantation is contraindicated in patients with cardiovascular issues, poor oral hygiene, or low motivation, as these factors could compromise the success of the procedure 3.

While autotransplantation is not yet a widely performed and underestimated procedure, it presents a viable alternative to dental implants and prosthetics due to its cost-effectiveness, simplicity, and ability to achieve aesthetically and functionally satisfying results. A unique advantage of autotransplantation is its potential to preserve the quantity and quality of alveolar bone including the surrounding soft tissue, which is essential for future dental health. The continuous improvement of techniques to increase autotransplantation success may expand the applicability of this treatment. One promising area of exploration involves using platelet-rich plasma (PRP), which is rich in growth factors, to enhance the success of autotransplantation, particularly in teeth with incomplete root formation.

Research has demonstrated that the stage of root development is a key factor influencing transplant success. Studies indicate that revascularization success rates reach 90-100% in teeth with open apices, whereas teeth with fully developed roots have higher rates of transplant failure 5. Therefore, it is crucial to explore methods for reducing complications such as ankylosis, pulpal necrosis, and tooth loss, which are commonly associated with post-transplant outcomes in mature teeth. Applying PRP to the recipient site during surgery may offer a means to minimize these complications by promoting healing and integration.

In recent years, advancements in imaging technologies, such as cone-beam computed tomography (CBCT) and computer-assisted planning, have significantly enhanced the predictability and precision of dental autotransplantation procedures. Studies have reported that digital workflow integration, including the use of 3D-printed surgical guides and donor tooth replicas, can improve donor tooth fit, reduce extraoral time, and preserve periodontal ligament integrity, all of which are critical for long-term success 6, 7. Moreover, the concept of atraumatic extraction has been refined through the use of piezoelectric surgical instruments and periotomes, minimizing trauma to the donor tooth and surrounding structures 8. These innovations aim to overcome limitations historically associated with autotransplantation and broaden its applicability, particularly in mature teeth with limited regenerative potential. This study contributes to the evolving field by evaluating whether adjunctive biological enhancement via PRP can further optimize clinical outcomes when combined with contemporary atraumatic techniques.

This study aims to investigate the short-term efficacy of PRP in autotransplantation of teeth with both complete and incomplete root development. Specifically, it seeks to evaluate and compare PRP's effects on tooth vitality, periodontal probing depth, mobility, pain, and root resorption. By focusing on these parameters, this research intends to provide insights into optimizing autotransplantation techniques for minimal morbidity and enhanced clinical outcomes.

## Materials and Methods

This prospective, randomized cohort study was conducted at the Department of Oral and Maxillofacial Surgery, Faculty of Dentistry, Istanbul University in compliance with the principles outlined in the Helsinki Declaration on Human Rights. The ethical approval was obtained from Clinical Research Ethics Committee of Istanbul University, Faculty of Dentistry (2019/1-Rev/2). Prior to participation, all patients were thoroughly informed about the study, and written informed consent was obtained for participation and publication.

Patients who required tooth extraction due to caries, periodontal disease, or irreparable damage to the lower and upper molars, with suitable donor teeth available for autotransplantation, were included in the study. A total of 20 patients meeting inclusion criteria were divided into two main groups based on the radiological root development level of the tooth intended for transplantation: Group 1 consisted of patients with fully developed roots, and Group 2 included patients with 1/3 or 2/3 root development. Each main group was further randomized into two subgroups based on platelet-rich plasma (PRP) application. Randomization was performed on www.randomizer.org, assigning 10 participants from each main group to two protocols (Protocol 1: without PRP application; Protocol 2: with PRP application).

Patients aged 18-25 years with good oral hygiene, identified through clinical and radiological examinations at the Department of Oral and Maxillofacial Surgery, Faculty of Dentistry, Istanbul University, were included. Patients with systemic diseases, osteoporosis/osteopetrosis, undergoing chemotherapy/radiotherapy, taking bisphosphonates, with neurological disorders (e.g., epilepsy), substance abuse, pregnancy, or lack of compliance, as well as two patients whose procedures failed after the 6-month follow-up, were excluded.

The sample size was determined by a power analysis, which indicated that with a 95% confidence level and 80% power, a sample size of 5 per group was sufficient to detect a statistically significant difference in periodontal probing depth between the PRP and non-PRP groups at the 9-month follow-up (mean probing depths: PRP group = 4.056 ± 0.416, non-PRP group = 2.889 ± 0.676) (reference: Sharma A, Pradeep AR, 2011).

The 20 patients were allocated as follows:

- Group 1 (n=5): Fully developed root without PRP application

- Group 2 (n=5): Partially developed root without PRP application

- Group 3 (n=5): Fully developed root with PRP application

- Group 4 (n=5): Partially developed root with PRP application

The surgical site was disinfected with povidone-iodine, and local anesthesia (2% articaine hydrochloride with epinephrine 1:100,000) was administered. Preparation of the recipient site began with atraumatic tooth extraction if necessary, minimizing surgical trauma. Following extraction, the recipient site was prepared using surgical fissure and round burs under sufficient irrigation. After carefully extracting the donor tooth, it was placed into the prepared recipient site and adjusted to sit slightly below occlusal level to prevent occlusal interference. If required, adjustments were made with surgical burs. The donor tooth was temporarily stored in saline solution at room temperature, and care was taken not to damage the root surface, particularly the Hertwig's epithelial root sheath. Stabilization of the transplanted tooth was achieved using a horizontal eight mattress suture with 3.0 silk around the gingiva, ensuring the tooth was securely positioned. Postoperatively, a 5-day antibiotic and analgesic-anti-inflammatory regimen, as well as chlorhexidine mouthwash, was prescribed. Sutures were removed at the 1-week follow-up.

Approximately 2 cc of venous blood (2 tubes) was drawn from patients assigned to receive PRP, using vacutainer tubes without anticoagulant (Greiner Bio-One, GmbH, Austria). Blood samples were immediately centrifuged (MedifugeTM MF200, Silfradent srl, Italy) with a 27-minute program in the laboratory at Istanbul University. In the first step, samples were centrifuged at 3,500 rpm for 12 minutes, separating erythrocytes to the bottom layer, while plasma remained on top. In the second step, the plasma layer was centrifuged again at 3,000 rpm for 15 minutes, concentrating the platelets at the bottom, yielding PRP ready for application.

Patients underwent clinical and radiological follow-ups immediately post-transplantation, and at 1 week, 1, 3, 6, and 12 months. Assessments included:

1. Vitality Test: Assessed by a vitality meter to measure pulpal response to thermal changes; responses were recorded as positive (+) or negative (-).

2. Periodontal Probing Depth: Probing depth was measured with a periodontal probe at four sites (mesial, distal, lingual/palatal, buccal/labial), and the average value was recorded in millimeters (mm).

3. Tooth Mobility: Evaluated manually by applying force horizontally to the buccal and lingual surfaces using dental instruments; results were recorded as positive (+) or negative (-).

4. Pain Assessment: Measured using a 10 cm Visual Analog Scale (VAS), where 0 indicated no pain, 10 indicated the most severe pain, and 5 represented moderate pain. Patients marked their pain level on the scale.

5. Root Resorption/Development: Measured on periapical radiographs as the distance from the cemento-enamel junction to the apex of the mesial root in millimeters.

Non-vital teeth showing pathological changes at the 1-month follow-up received root canal therapy and were documented as non-vital (-). Two teeth, classified as failures, exhibited progressive root resorption and attachment loss post-transplantation.

## Results

There were no statistically significant differences in the average age or gender distribution between the control groups (Groups 1 and 2) and the PRP groups (Groups 3 and 4) (p=0.501, p=0.531). Additionally, no significant differences were observed between the control and PRP groups regarding the distribution of recipient sites and donor teeth (p=0.598, p=0.717).

Throughout the follow-up period at postoperative weeks 1, months 1, 3, 6, and 12, there were no statistically significant differences in vitality distribution between the control and PRP groups (p>0.05). Likewise, no significant differences in mobility distribution were observed between the control and PRP groups at these same time points (p>0.05) (Table 1).

Periodontal probing depth (mm) averages also did not differ significantly between the control and PRP groups at postoperative weeks 1 and months 1, 3, 6, and 12 (p>0.05). Within each group, there was no significant change in probing depth averages across these time points, with p=0.061 for the control groups and p=0.471 for the PRP groups.

When comparing pain levels at postoperative week 1, no significant difference was found between the control and PRP groups (p=1). Pain was not observed in either group at months 1, 3, 6, and 12. Root length averages between the control and PRP groups showed no statistically significant differences across all postoperative time points (p>0.05) (Table 2).

Within the control groups (Groups 1 and 2), a significant change in root length averages was observed from week 1 through month 12 (p=0.004). Root length averages were significantly higher at week 1 compared to months 1, 3, 6, and 12 (p=0.042, p=0.007), at month 1 compared to months 3, 6, and 12 (p=0.009, p=0.006), at month 3 compared to months 6 and 12 (p=0.029, p=0.009), and at month 6 compared to month 12 (p=0.029).

Similarly, in the PRP groups (Groups 3 and 4), root length averages exhibited statistically significant changes over the follow-up period (p=0.001). Root length averages at week 1 were significantly higher than at months 3, 6, and 12 (p=0.003, p=0.005), at month 1 compared to months 3, 6, and 12 (p=0.001, p=0.0001), at month 3 compared to months 6 and 12 (p=0.005, p=0.0001), and at month 6 compared to month 12 (p=0.011) (Table 3).

Regarding resorption, inflammatory resorption was observed in 11 out of 20 teeth, while replacement resorption was identified in 5 teeth. In the remaining 4 teeth, no significant inflammatory or replacement resorption was detected.

## Discussion

Autotransplantation offers a biological viable alternative to implants and prosthetics for addressing tooth deficiencies, with the advantage of preserving the alveolar crest shape, maintaining physiological tooth mobility, and supporting stomatognathic system functions at a lower cost. Reported success rates for autotransplantation range between 74% and 100% in literature, and success is influenced by numerous factors, including patient age, root development stage, donor-recipient site compatibility, and atraumatic surgical handling 1, 9, 10. Notably, the stage of root development is a critical determinant for transplantation success. As studies by Slagsvold and Bjercke (1974) and Schwartz et al. highlighted, teeth with open apices exhibit higher success rates due to their increased potential for revascularization, especially in cases where root development is between half and three-quarters 11, 12, 13. In contrast, teeth with fully developed roots are at a higher risk for complications like inflammatory root resorption and ankylosis, often linked to periodontal ligament (PDL) damage during surgery 2, 14, 15.

This study aimed to assess the effects of platelet-rich plasma (PRP) on short-term outcomes in autotransplanted teeth, examining indicators such as vitality, probing depth, mobility, pain, and root resorption. Although PRP has been recognized for its regenerative potential in various fields, including orthopedics and dentistry, our results indicated no statistically significant differences between PRP-treated and control groups across these outcomes. This finding suggests that PRP's regenerative effects may be limited under certain conditions in dental autotransplantation, especially when the transplanted teeth have mature roots.

Molar teeth are the most commonly extracted teeth due to caries or periodontal disease, making them a primary focus in autotransplantation studies 16. In our study, we included patients who required molar extractions due to extensive caries, periodontal disease, or irreparable damage and who also had suitable donor teeth. The main challenge in autotransplantation is the availability of an appropriate donor tooth. Kim et al. reported that ideal donor teeth for transplantation are those with sufficient root length and volume, easy extraction potential, and without periodontal problems 17.

Ankylosis-related resorption, a common cause of failure in tooth autotransplantation, is more prevalent among older patients 18. To minimize the risk of ankylosis in our study, we utilized a horizontal eight mattress suture technique to provide semi-fixation instead of rigid stabilization. This approach aligns with Tsukiboshi's (2023) protocol, where suture-based fixation is preferred to control mobility while reducing both operative time and patient discomfort 19.

Healing of periodontal tissues involves competitive regeneration between periodontal ligament fibroblasts and osteoblasts, reported to continue for up to 4-16 weeks post-transplantation 20. Our study's objective was to evaluate the effectiveness of PRP, which accelerates periodontal healing and increases local growth factor concentration, during this critical 16-week healing period in dental autotransplantation. PRP is an autologous blood component derived from the patient's blood and enriched with platelets and hyperphysiological levels of growth factors. Normally, the cellular component of plasma consists of 93% erythrocytes, 6% platelets, and 1% leukocytes; however, PRP contains 3-5 times more platelets than whole blood, amplifying its regenerative potential 21, 22.

The findings from the existing literature also align with these results. In a systematic review by Machado et al. (2021), autotransplantation success rates ranged from 75.3% to 91%, with factors such as root development, surgical technique, and site compatibility influencing outcomes 23. Studies like those by Andreasen and Kahler (2018) also stress that the revascularization potential of immature roots significantly contributes to better outcomes, as these roots have an inherent ability for cellular regeneration, a factor that may limit the benefits of PRP in cases with fully developed roots 24. Additionally, Tan et al. (2022) emphasized the importance of atraumatic surgical techniques, which were crucial in our study as well, where the handling of the periodontal ligament was a key factor in the success of the procedure 25.

Emerging evidence over the past five years has underscored the growing role of technology-driven planning and biologically oriented approaches in improving autotransplantation outcomes. Ong et al. (2023) demonstrated that incorporating CBCT-based planning and 3D-printed donor replicas significantly decreased surgical time and improved root adaptation, especially in cases involving fully formed roots where precision is paramount 26. Furthermore, the use of stem cell-derived scaffolds and platelet concentrates beyond PRP -such as concentrated growth factors (CGF) and injectable platelet-rich fibrin (i-PRF)- have been explored to modulate the healing environment and support periodontal regeneration 27-31. Compared to these advanced techniques, the single-use PRP application used in this study may provide limited stimulation of healing pathways, particularly in the context of mature teeth with diminished vascular and cellular activity. Therefore, while PRP remains a safe and autologous adjunct, its clinical value may be enhanced through multimodal regenerative strategies and integration into digitally guided surgical workflows. These considerations emphasize the need for future trials employing synergistic applications of biomaterials, image-guided surgery, and repeat-dose biologics to fully exploit the regenerative potential of autotransplantation.

Vitality and mobility are essential metrics for autotransplantation success, reflecting the tooth's reintegration into the recipient site and the health of the periodontal ligament. In our study, both PRP-treated and control groups showed similar outcomes in these areas, which suggests that PRP may not significantly enhance early reintegration. This aligns with existing literature highlighting the importance of root development stage, as immature roots support better outcomes due to active revascularization potential 11, 12. The limited impact of PRP on vitality and mobility in our findings may be due to the innate regenerative capabilities of the PDL, particularly in immature teeth that already possess active cellular regeneration processes. Furthermore, the absence of significant differences in probing depth underscores the importance of careful surgical handling and preservation of the periodontal ligament to prevent complications such as ankylosis and root resorption 14, 15.

Our findings also revealed that PRP did not significantly affect pain reduction in the early postoperative period. While PRP contains anti-inflammatory factors, its role in immediate pain modulation may be limited in dental autotransplantation, where postoperative inflammation is typically minimal 32. Pain in autotransplantation appears more dependent on surgical technique and postoperative care protocols, rather than on PRP. Further research is warranted to explore alternative pain management strategies that could be complementary in this context.

Previous studies have often reported an increase in root length in autotransplanted teeth, especially in cases where the teeth have not yet completed root development 23. This increase is largely attributed to the regenerative potential of young, developing teeth and the favorable healing response supported by growth factors and cell proliferation. However, in our study, inflammatory resorption was observed instead of the anticipated root length increase in a notable proportion of cases. This finding may be explained by the relatively older age of our patient population and the near-complete root development of the transplanted teeth. As teeth approach the final stages of root maturation, their regenerative capacity diminishes, leading to an increased likelihood of inflammatory resorption rather than continued growth. The reduced healing potential in mature teeth, coupled with the higher incidence of inflammatory processes in older individuals, may thus account for the prevalence of inflammatory resorption observed in our study. Our findings suggest that patient age and root development stage are critical factors in predicting the resorption type and may play a pivotal role in influencing the outcomes of autotransplantation procedures.

In this study root resorption did not show significant differences between the PRP and control groups in this study. This suggests that PRP may not directly inhibit resorptive processes. As literature indicates, the prevention of root resorption involves complex cellular interactions, which may not be sufficiently addressed by PRP's growth factors alone 2, 15, 31. Although PRP has been reported to modulate inflammation and support early wound healing, these effects may not extend significantly to mature roots where regenerative potential is limited. Future studies might explore the impact of repeated or prolonged PRP applications to investigate its potential in providing protection against resorption. Studies like those by Ong et al. (2023) suggest that repeated applications of PRP, especially in high-risk cases, could be worth investigating to explore its protective effects on resorptive processes 26.

Several limitations should be acknowledged in this study. The follow-up period of 12 months may not capture the full spectrum of PRP's effects on autotransplantation success, especially regarding late-onset resorption or ankylosis. Longer follow-up periods are recommended to observe potential late-stage complications 1. Additionally, PRP was applied only once, which may not fully leverage its regenerative capacity. Future studies with higher concentrations of specific growth factors within PRP or repeated applications could provide further insights into its therapeutic role. Finally, larger sample sizes and subgroup analyses by root development stage may be necessary to clarify PRP's impact, particularly for high-risk cases or teeth with incomplete root formation.

In conclusion, this study found that PRP application in tooth autotransplantation did not yield statistically significant differences in short-term outcomes for vitality, probing depth, mobility, pain, or root resorption. While PRP presents an autologous, cost-effective, and readily prepared option with known regenerative benefits, its impact on short-term outcomes in dental autotransplantation appears limited. Further studies with refined PRP protocols, extended follow-up periods, and focused analyses on cases needing enhanced periodontal or pulpal support, particularly in mature teeth, are necessary to better elucidate PRP's clinical utility in this field.

## Figures and Tables

**Figure 1:**
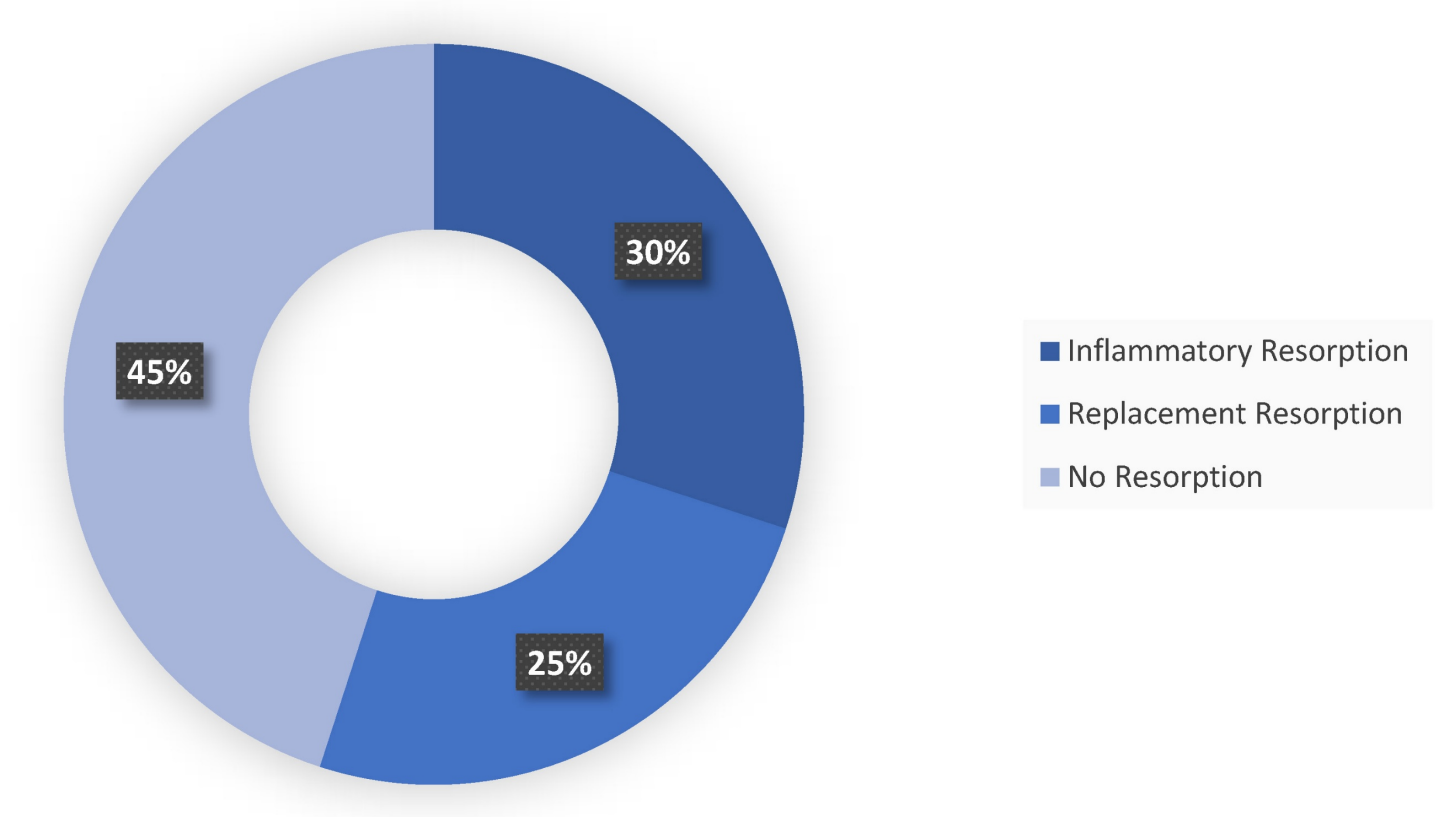
Graph Showing the Distribution of Root Resorption Types.

**Table 1:** Comparison of Postoperative Findings Using Chi-Squared Test

Vitality				Control Groups				PRP Groups			p
Week 1		(-)		9		90,00%		6		60,00%	0,121
		(+)		1		10,00%		4		40,00%	
Month 1		(-)		8		80,00%		5		50,00%	0,160
		(+)		2		20,00%		5		50,00%	
Month 3		(-)		8		80,00%		5		50,00%	0,160
		(+)		2		20,00%		5		50,00%	
Month 6		(-)		8		80,00%		5		50,00%	0,160
		(+)		2		20,00%		5		50,00%	
Month 12		(-)		8		80,00%		5		50,00%	0,160
		(+)		2		20,00%		5		50,00%	
Mobility			Control Groups				PRP Groups				p
Week 1	(-)		5		50,00%		3		30,00%		0,361
	(+)		5		50,00%		7		70,00%		
Month 1	(-)		7		70,00%		6		60,00%		0,639
	(+)		3		30,00%		4		40,00%		
Month 3	(-)		10		100,00%		10		100,00%		-
Month 6	(-)		10		100,00%		10		100,00%		-
Month 12	(-)		10		100,00%		10		100,00%		-
											

**Table 2:** Comparison of Postoperative Findings Using Independent T-Test and Paired Analysis of Variance

Periodontal Depth (mm)	Control Groups	PRP Groups	p*
Week 1	3,08±0,88	2,83±0,80	0,515
Month 1	2,43±0,90	2,45±0,89	0,951
Month 3	2,25±0,79	2,50±0,53	0,416
Month 6	2,20±0,42	2,60±0,70	0,139
Month 12	2,60±0,52	2,40±0,70	0,476
p‡	0,061	0,471	
Pain	Control Groups	PRP Groups	p
Week 1	0,30±0,67	0,30±0,67	1
Month 1	0±0	0±0	
Month 3	0±0	0±0	
Month 6	0±0	0±0	
Month 12	0±0	0±0	
p	-	-	
Root Length	Control Groups	PRP Groups	p
Week 1	10,07±1,13	9,08±1,65	0,135
Month 1	9,37±1,27	8,37±1,86	0,178
Month 3	9,14±1,33	7,92±1,86	0,109
Month 6	9,05±1,34	7,76±1,88	0,094
Month 12	9,01±1,35	7,68±1,89	0,087
p	0,004	0,001	

**Table 3:** Evaluation the Differences Between the Means Using Newman Keuls Multiple Comparison Test

Post-operative	Control Groups	PRP Groups
Week 1/ Month 1	0,042	0,062
Week 1/ Month 3	0,011	0,005
Week 1/ Month 6	0,008	0,003
Week 1/ Month 12	0,007	0,003
Month 1/ Month 3	0,009	0,001
Month 1/ Month 6	0,008	0,0001
Month 1/ Month 12	0,006	0,0001
Month 3/ Month 6	0,029	0,005
Month 3/ Month 12	0,009	0,0001
Month 6/ Month 12	0,037	0,011
